# Distribution and amplification of interstitial telomeric sequences (ITSs) in Australian dragon lizards support frequent chromosome fusions in Iguania

**DOI:** 10.1371/journal.pone.0212683

**Published:** 2019-02-22

**Authors:** Kornsorn Srikulnath, Bhumika Azad, Worapong Singchat, Tariq Ezaz

**Affiliations:** 1 Institute for Applied Ecology, Faculty of Science and Technology, University of Canberra, Bruce, Australian Capital Territory, Australia; 2 Laboratory of Animal Cytogenetics and Comparative Genomics (ACCG), Department of Genetics, Faculty of Science, Kasetsart University, Bangkok, Thailand; Universita degli Studi di Roma La Sapienza, ITALY

## Abstract

Telomeric sequences are generally located at the ends of chromosomes; however, they can also be found in non-terminal chromosomal regions when they are known as interstitial telomeric sequences (ITSs). Distribution of ITSs across closely related and divergent species elucidates karyotype evolution and speciation as ITSs provide evolutionary evidence for chromosome fusion. In this study, we performed physical mapping of telomeric repeats by fluorescence *in situ* hybridisation (FISH) in seven Australian dragon lizards thought to represent derived karyotypes of squamate reptiles and a gecko lizard with considerably different karyotypic feature. Telomeric repeats were present at both ends of all chromosomes in all species, while varying numbers of ITSs were also found on microchromosomes and in pericentromeric or centromeric regions on macrochromosomes in five lizard species examined. This suggests that chromosomal rearrangements from ancestral squamate reptiles to Iguania occurred mainly by fusion between ancestral types of acrocentric chromosomes and/or between microchromosomes, leading to appearance of bi-armed macrochromosomes, and in the reduction of microchromosome numbers. These results support the previously proposed hypothesis of karyotype evolution in squamate reptiles. In addition, we observed the presence of telomeric sequences in the similar regions to heterochromatin of the W microchromosome in *Pogona barbata* and *Doporiphora nobbi*, while sex chromosomes for the two species contained part of the nucleolar organiser regions (NORs). This likely implies that these ITSs are a part of the satellite DNA and not relics of chromosome fusions. Amplification of telomeric repeats may have involved heterochromatinisation of sex-specific W chromosomes and play a role in the organisation of the nucleolus.

## Introduction

Positive correlation of karyotype diversity and species richness is found in squamate reptiles [1]. Both macro- and microchromosomes are commonly found in Scincoidea and Episquamata, exclusive of Lacertidae; by contrast, only few or no microchromosomes are found in Lacertidae and most species of Gekkota [2]. Comparative genome analysis among squamate reptiles, using chicken as a reference species, identified high levels of conserved synteny across species, allowing delineation of the process of chromosomal rearrangements over 200 million years [3–13]. All macrochromosomes of Toxicofera species (snakes, anguimorphid lizards and iguanian lizards) are highly conserved in lacertid and gekkonid lizards, which have variable karyotypes. Nine of seventeen macrochromosome pairs of sand lizard (*Lacerta agilis*) karyotype (2n = 38: 36 macrochromosomes and 2 microchromosomes) showed conserved synteny with ten chromosome pairs of the Hokou gecko (*Gekko hokouensis*) karyotype (no microchromosomes) and macrochromosomes and/or macrochromosome segments of Toxicofera species [8,9]. This suggests that series of fusion-fission events between macrochromosomes or other microchromosomes has resulted in the evolution of diversified/variable karyotypes in squamate reptiles [8,9,12].

The karyotype of iguanian lizards (Iguania) is highly conserved with a diploid complement of 36 including six pairs of macrochromosomes and twelve pairs of microchromosomes [2,14]. Karyotypes in Iguania is proposed to have evolved by repeated fusion between macrochromosomes and/or other microchromosomes, leading to the origin of bi-armed macrochromosomes and reduction of microchromosomes [6–9,12]. With few exceptions, most of the Australian dragon lizards exhibit 2n = 32, including six pairs of macrochromosomes and ten pairs of microchromosomes, implying that repeated fusion predominantly occurred in this group [2,14]. Dynamic transitions of sex determination are also found in the same evolutionary clade, although their karyotype is highly conserved [15–18]. Our previous chromosome mapping indicated the occurrence of simple translocation between macrochromosomes and sex microchromosomes or between macrochromosomes and microchromosomes in Australian dragon lizards ([11], Matsubara et al. unpublished data). This suggests that change in chromosome structure affected the transition of sex determination mode and make this lineage an excellent research model for evolution of genome, chromosomes and sex chromosomes. Interestingly, change in chromosome structure, especially chromosome fusion often relates to telomeric repeats [19,20]; thus, it is extremely important to understand how telomeric repeats relate to chromosomal rearrangement in this lineage.

Vertebrate chromosome ends are capped by telomeres that physically protect them from DNA repair and recombination, maintain chromosome stability and integrity, and control replicative lifespan [21,22]. This structure comprises nucleoprotein complexes associated with tandem telomeric DNA repeats (TTAGGG), widely recognised as a remarkable evolutionary conservation feature [21,22]. However, the distribution of these sequences at non-telomeric sites, known as interstitial telomeric sequences (ITSs) or interstitial telomeric repeats (ITRs) was also observed in several lineages [22]. ITSs can be classified into two different types according to their organisation as short ITSs (s-ITSs) and heterochromatic ITSs (het-ITSs) [19]. The s-ITSs exist as short sized telomeric DNA involving mechanisms of DNA repair, while het-ITSs are large stretches of telomeric sequences located mainly in centromeric or pericentromeric heterochromatic regions or euchromatic regions within the chromosome arms. The het-ITSs are considered to be relics of chromosomal rearrangements in the evolutionary process involving fusion or inversion which occurred within derived karyotypes. In squamate reptiles, the presence of ITSs is frequently observed in Iguania, which is supposed to have a derived karyotype from other squamate reptiles [6,12,23,24]. To test this hypothesis and to better characterise Iguanian karyotype evolution, distribution of telomeric sequences in Australian dragon lizards was analysed together with a gecko lizard (*Hemidactylus frenatus*) with largely different karyotypes as the outgroup. The significance of telomeric distribution was discussed.

## Materials and methods

### Animal and chromosome preparation

Seven dragon lizard species containing both male and female individuals and both male and female gecko lizard species were examined. Detailed information is presented in Table 1. Euthanasia and sexing were performed as described in Ezaz et al. [16]. Briefly, animals were euthanised by an intraperitoneal injection of sodium pentobarbitone at a concentration of 150 mg/g body weight. Phenotypic sex was determined on the basis of external morphology by hemipenes eversion [25] and by internal examination of gonadal morphology. All experiments were performed with the approval of the University of Canberra and Kasetsart University Animal Experimentation Ethics Committee (CEAE 11/07 and ACKU61-SCI-021). Metaphase chromosomes were prepared either from short-term culture of whole blood or from fibroblast cell lines as described by Ezaz et al. [15,16] and Srikulnath et al. [26]. Chromosome slides were treated with 100 μg/ml RNase for 1 h at 37°C and then rinsed three times in 2 × SSC.

**Table 1:** List of species, collection sites and their interstitial telomeric sequence (ITS) distribution.

Species	Chromosome number	Sex determination^1^	Collection locality^2^	ITS distribution		Number of animals used (female+male)	Reference
				Female	Male		
*Diporiphora nobbi*	2n = 32	GSD (ZW)	Vic	Chromosome 1 and W chromosome	Chromosome 1	1+1	this study
*Pogona vitticeps*	2n = 32	GSD (ZW)	NSW	W chromosome and 8 microchromosomes	8 microchromosomes	1+1	[11]
*Pogona barbata*	2n = 32	GSD (ZW)	ACT	Sex chromosomes and 2 microchromosomes	Z chromosome and 2 microchromosomes	1+1	this study
*Amphibolurus norrissi*	2n = 32	GSD	Vic	All macrochromosomes and 4 microchromosomes	All macrochromosomes and 4 microchromosomes	1+1	this study
*Amphibolurus muricatus*	2n = 32	TSD	ACT	All macrochromosomes and 2 microchromosomes	All macrochromosomes and 2 microchromosomes	1+1	this study
Ctenophorous fordi*	2n = 32	GSD, ZW	-	2 microchromosomes	2 microchromosomes	1+1	this study
*Physignathus leseuirii*	2n = 36	TSD	ACT	2 microchromosomes	2 microchromosomes	1+1	this study
*Hydrosaurus pustulatus*	2n = 36	unknown	Taronga Zoo	ND^3^	Chromosome 1	0+1	this study
*Hemidactylus frenatus*	2n = 40	XY	Bangkok	-	-	1+1	this study

^1^Sex determination; GSD: genotypic sex determination; TSD: temperature dependent sex determination

^2^Collection locality; ACT: Australian Capital Territory; NSW: New South Wales; Vic: Victoria

*pet trade.

^3^No data.

### Telomere peptide nucleic acid probe

Telomeric mapping was performed as described previously [27]. Briefly, 10 μl of hybridisation mixture containing 70% formamide, 0.3 μg/ml Cy_3_-(CCCTAA)_3_ peptide nucleic acid (PNA) probe (Biosynthesis, Inc., Texas) and 1× Denhardt’s solution in 10 mM Tris pH 7.2 were added to the slide under a coverslip and sealed with rubber cement. The DNA was denatured by heating for 3 min at 80°C. After hybridisation for 2 h at 37°C in a humidified chamber, the slides were washed at room temperature with 70% formamide, 1% BSA, 10 mM Tris pH 7.2 (2 times for 15 min) and then with 0.1 M Tris, 0.15 M NaCl, pH 7.5 containing 0.08% Tween-20 (3 times for 5 min). The slides were then dehydrated through an ethanol series (1 min in each of a 70%, 90% and 100% solution), air dried, stained with DAPI (4′, 6′-diamidino-2-phenylindole) (50 μg/ml DAPI solution in 2 × SSC) for 45 s at room temperature and mounted with Vectashield (Vector Laboratories, Inc., Burlingame, CA, USA). Images were captured using a Zeiss Axio Scope A1 epifluorescence microscope fitted with a high-resolution microscopy camera AxioCam MRm Rev. 3 (Carl Zeiss Ltd.) and analysed using AxioVision v4.8.1 software or ISIS Fluorescence Imaging System (MetaSystems, Altlussheim, Germany).

## Results

**Fig 1:**
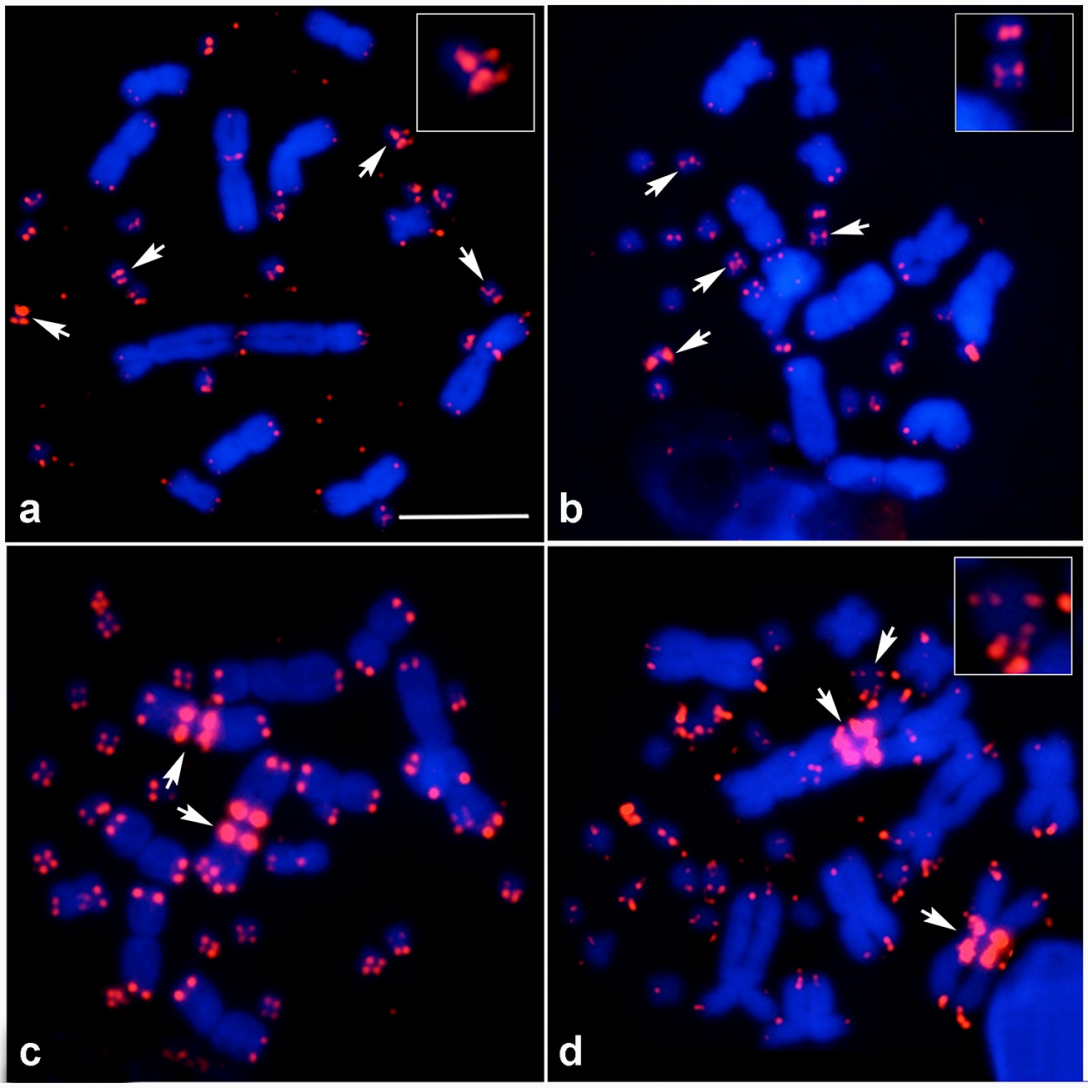
Chromosomal locations of (CCCTAA)n sequences in *Pogona barbata* and *Diporiphora nobbi*. Hybridisation pattern of Cy3-labelled CCCTAA repeats (red) in male *P*. *barbata* (a), female *P*. *barbata* (b), male *D*. *nobbi* (c) and female *D*. *nobbi* (d). Arrows indicate signals of interstitial telomeric sites (ITSs). The square box indicates the enlarged chromosome containing ITSs. Scale bar represents 10 μm.

**Fig 2:**
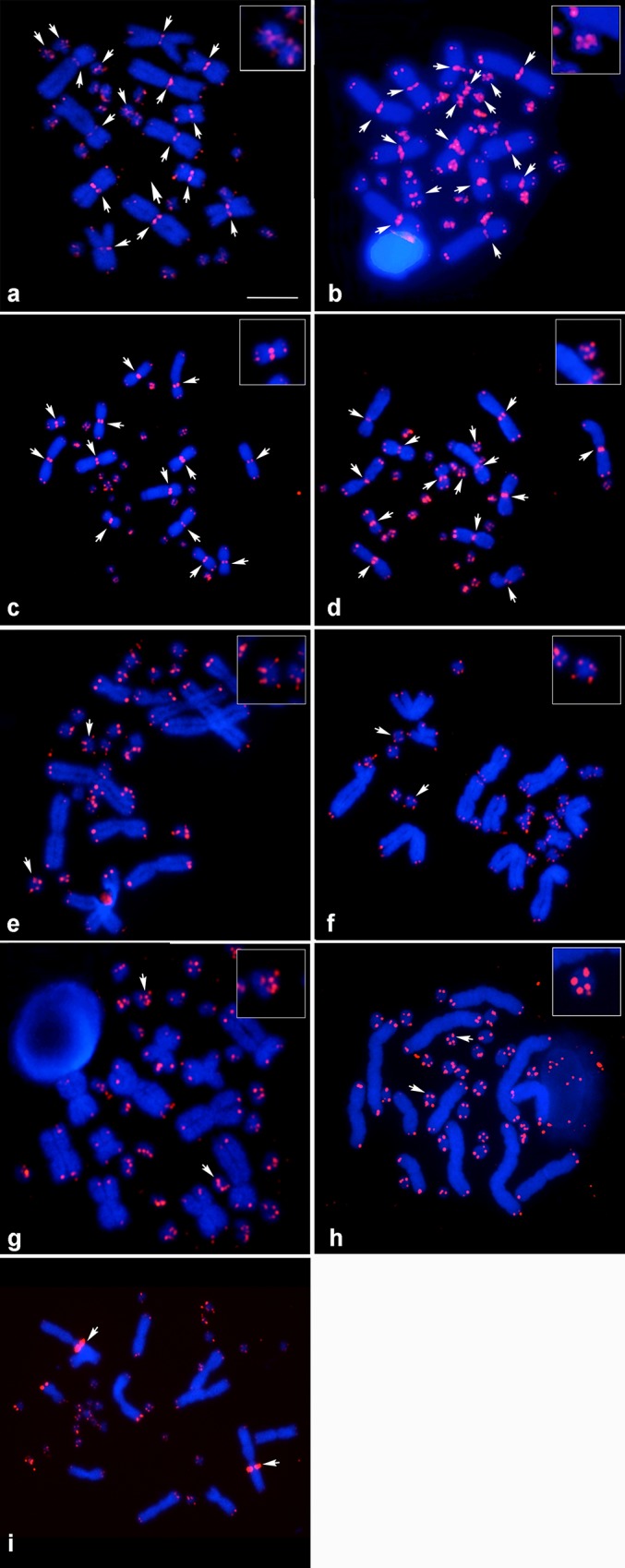
Chromosomal locations of (CCCTAA)n sequences in *Amphibolurus norrissi*, *Amphibolurus muricatus*, *Ctenophorous fordi*, *Physignathus leseuirii* and *Hydrosaurus pustulatus*. Hybridisation pattern of Cy3-labelled CCCTAA repeats (red) in male *A*. *norrissi* (a), female *A*. *norrissi* (b), male *A*. *muricatus* (c), female *A*. *muricatus* (d), male *C*. *fordi* (e), female *C*. *fordi* (f), male *P*. *leseuirii* (g), female *P*. *leseuirii* (h) and male *H*. *pustulatus* (i). Arrows indicate signals of interstitial telomeric sites (ITSs). The square box indicates the enlarged chromosome containing ITSs. Scale bar represents 10 μm.

**Fig 3:**
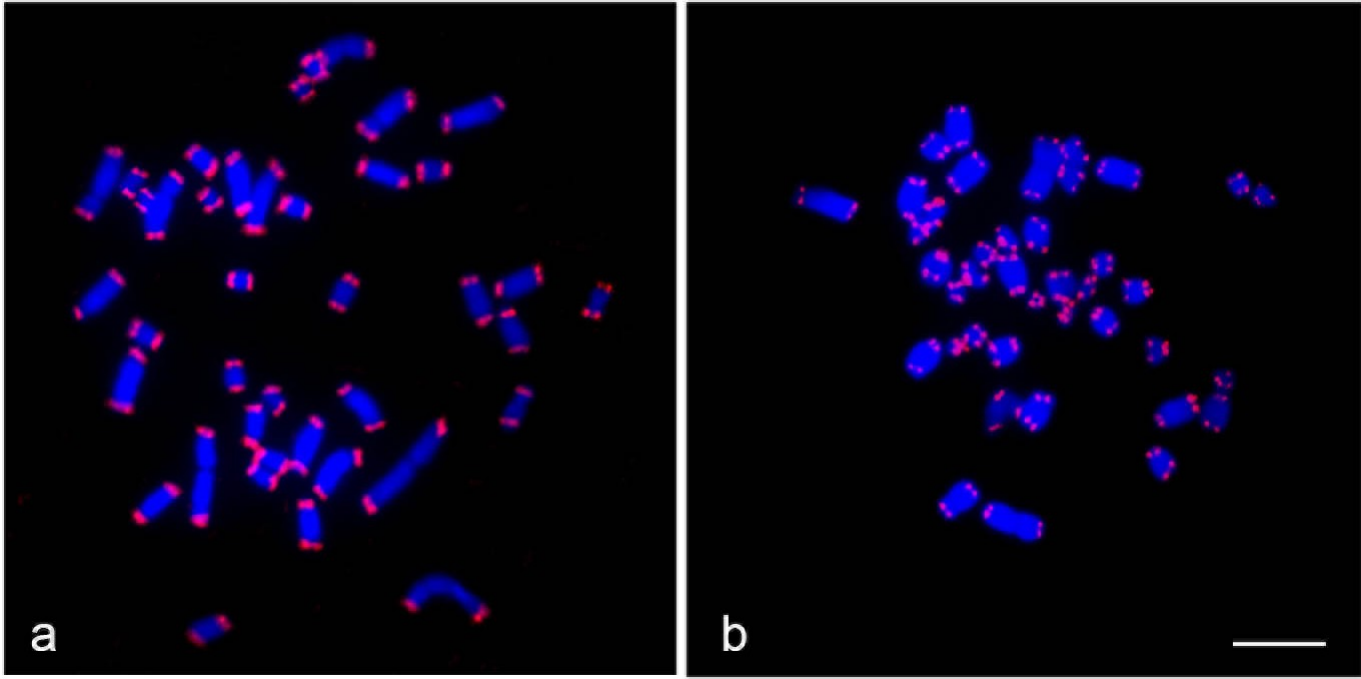
Chromosomal locations of (CCCTAA)n sequences in *Hemidactylus frenatus*. Hybridisation pattern of Cy3-labelled CCCTAA repeats (red) in male *H*. *frenatus* (a), female *H*. *frenatus* (b). Scale bar represents 10 μm.

Fluorescence hybridisation signals of the PNA telomeric probe Cy_3_-(CCCTAA)_3_ were observed at the telomeric ends of all chromosomes in seven dragon lizards and the gecko lizard (Figs 1–3). The hybridisation signals were weak on macrochromosomes; by contrast, high intensity signals were observed on almost all microchromosomes in the seven dragon lizards. ITSs were observed in all species examined. In *Pogona barbata*, ITSs were found on several microchromosomes and sex microchromosomes (Z and/or W chromosomes), while ITSs were localised to one microchromosome pair of *Ctenophorous fordi* and *Physignathus leseuirii* (Table 1). ITSs were localised to the W microchromosome and pericentromeric regions of chromosome 1 in *Diporiphora nobbi* but only near the pericentromeric region of chromosome 1 in *Hydrosaurus pustulatus*. By contrast, ITSs were detected in the centromeric regions of all macrochromosomes and two microchromosome pairs in *Amphibolurus norrissi* and *A*. *muricatus* (Fig 2). No ITSs were found on the gecko lizard *H*. *frenatus* (Fig 3).

## Discussion

**Fig 4:**
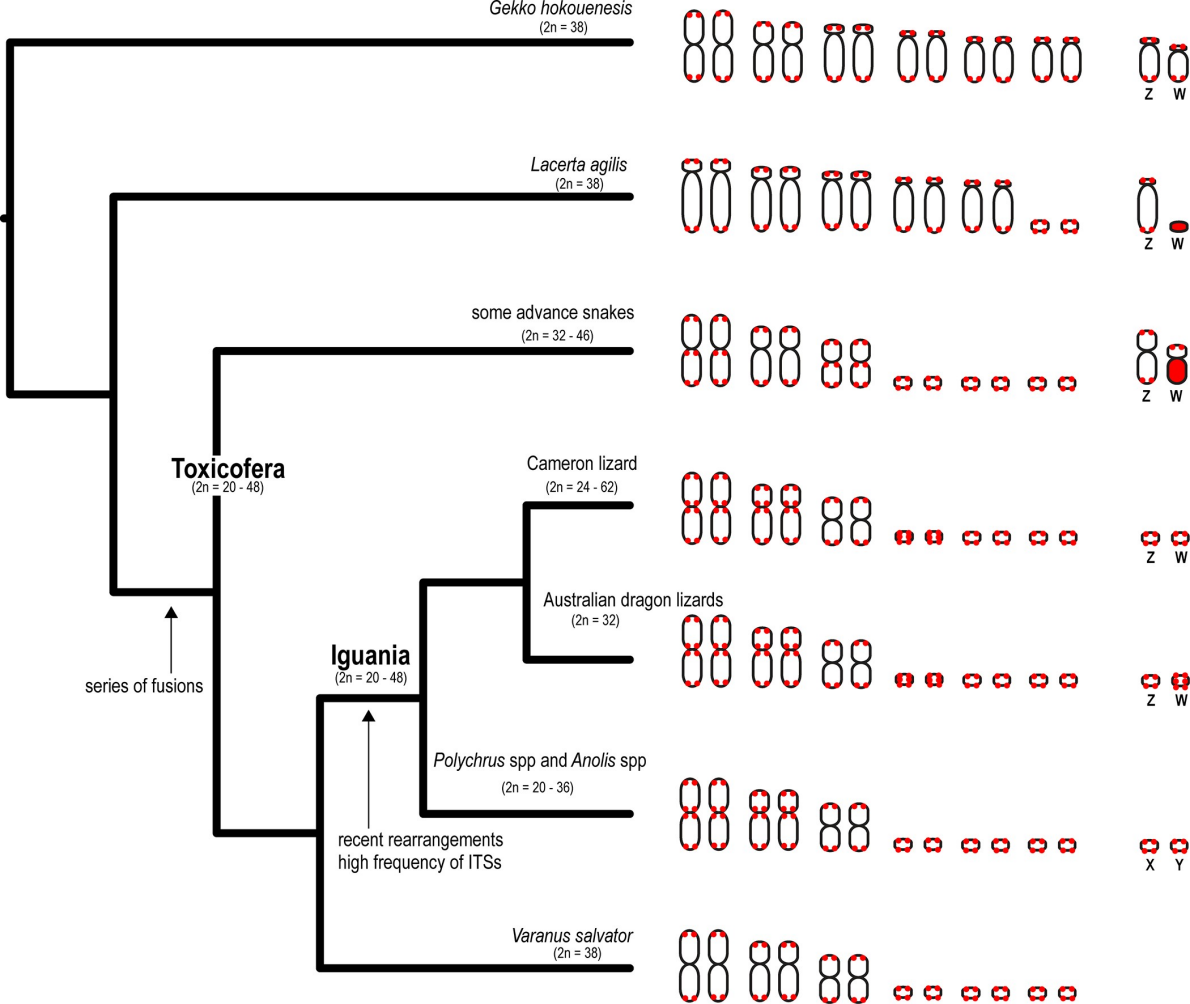
Schematic representation of the distribution of interstitial telomeric sites (ITSs) in centromeric or pericentromeric regions on chromosomes and amplification of telomeric repeats on sex chromosome in lizards and snakes. Phylogeny was partially derived from Pyron et al. [39]. Distribution of ITSs and telomeric repeat amplifications of lizards and snakes were also obtained from Young et al. [11], Matsubara et al. [37], Augstenová et al. [34], Rovatsos et al. [23,30] and Srikulnath et al. [7,9].

Comparative chromosome maps of squamate reptiles suggest that chromosome fusion between macrochromosomes or other microchromosomes occurred in iguanian lizards, resulting in all macrochromosomes being bi-armed and in the reduction of microchromosome numbers [6–9,12], while cross-species chromosome painting analysis revealed several conserved syntenies among gecko lizard chromosomes (*Gekko* and *Hemidactylus*) [28]. In our study, highly intense signals were observed on almost all microchromosomes in the seven dragon lizards but not on macrochromosomes, suggesting that telomeric repeats were amplified site-specifically. We also observed ITSs in pericentromeric or centromeric regions of all bi-armed macrochromosomes in *A*. *norrissi* and *A*. *muricatus*, similar what has been reported in several other iguanian lizards, such as *Polychrus acutirostris*, *P*. *marmoratus*, *Anolis distichus* and *A*. *equestris* and [23,29,30]. Iguanian lizards and snake chromosome 1 correspond to two acrocentric chromosomes of lacertid lizards and two chromosome arms of different bi-armed chromosomes in gekkonid lizards [6–9,11,12,31]. ITSs observed in pericentromeric regions of chromosome 1 in *D*. *nobbi*, *H*. *pustulatus*, and two species of *Amphibolurus* also show cytogenetic evidence that chromosome 1 in iguanian lizard is derived from centric fusion between the ancestral acrocentric chromosomes. Several pericentric inversions have been discovered in macrochromosomes of *Anolis carolinensis* [32] which may represent remnants of ancestral intrachromosomal rearrangements that occurred in the lineage. Additionally, ITSs were found in several microchromosomes within our study species with the exception of *H*. *pustulatus*, suggesting likely chromosome fusions between microchromosomes. However, several events involving chromosome fusion were not observed for ITSs in some iguanian lizard species. This suggests that the loss of telomeric repeats in interstitial regions have resulted from recombination events over evolutionary time [33], leading to a variety of ITS distribution in different iguanian lizards. Incidence of ITS distribution in iguanian lizards is higher than those of snakes and anguimorphid lizards (Toxicofera species) [23,30]. This suggests that these recently rearranged chromosomes in iguanian lizards have retained the relics of former telomeres on the segments when compared with those of snakes and anguimorphid lizards that probably occurred long evolutionary time ago (Fig 4). However, total number of species tested for the presence of ITSs remains small in snakes and anguimorphid lizards [7,23,34,35]. Therefore, chromosome mapping of telomeric repeats will be required in more snakes and anguimorphid lizards to clarify these finding as conclusive evidence.

Telomeric DNA can also contain repeat elements similar to centromeric repeat elements [36]. This suggests that ITSs on the centromeres may result from extensive amplification of telomere-like satellite DNAs within centromere. Molecular cloning of centromeric satellite DNA in Australian dragon lizards will be required to test whether similar amplification has also occurred within this group. Additionally, the heterochromatic W microchromosome showed large amplification of telomeric repeats in *P*. *barbata* and *D*. *nobbi*. A similar pattern was observed in *P*. *vitticeps* as closely related species with *P*. *barbata* [11]. Such amplification of telomeric repeats in the heterochromatic region on W chromosomes has been reported in several caenophidian snakes and other squamate reptiles [11,34,35,37,38]. These telomeric repeats are probably considered as a regular component of satellite DNA in heterochromatin. This suggests that amplification of repeat sequences probably involved heterochromatinisation of sex-specific chromosomes, particularly W chromosomes in species *D*. *nobbi*, *P*. *barbata* and *P*. *vitticeps*. The W sex chromosomes of *D*. *nobbi* and *P*. *barbata* also contained part of the nucleolar organiser regions (NORs) in addition to chromosome 2 [Matsubara et al. unpublished data]. Such ITSs showing co-localisation with NOR have also been reported in butterfly lizard chromosomes [20,26]). This suggests the possibility that NORs are associated with telomeric sequences which may play a role in the organisation of the nucleolus [38].

The karyotypic variability in Australian dragon lizards allowed examination of ITS relationships with chromosomal rearrangements, heterochromatin and NORs. Different patterns of ITSs may be due to the age of chromosomal rearrangements. Further studies involving chromosome mapping of telomeric repeats as well as satellite DNA in more representative species from divergent taxa are required to better understand evolutionary origins and distributions of ITS in squamate reptiles.
